# Association of immunity markers with the risk of incident frailty: the Rugao longitudinal aging study

**DOI:** 10.1186/s12979-021-00257-6

**Published:** 2022-01-03

**Authors:** Hui Zhang, Meng Hao, Zixin Hu, Yi Li, Xiaoyan Jiang, Jiucun Wang, Li Jin, Zuyun Liu, Xiaofeng Wang, Xuehui Sun

**Affiliations:** 1 Human Phenome Institute, Fudan University, Shanghai, China; 2 National Clinical Research Center for Aging and Medicine, Huashan Hospital, Fudan University, Shanghai, China; 3 State Key Laboratory of Genetic Engineering, Collaborative Innovation Center for Genetics and Development, School of Life Sciences, Fudan University, Shanghai, China; 4 Fudan Zhangjiang Institute, Shanghai, China; 5 Key Laboratory of Arrhythmias, Ministry of Education, Department of Pathology and Pathophysiology, School of Medicine, Tongji University, Shanghai, China; 6 Department of Big Data in Health Science, School of Public Health and the Second Affiliated Hospital, Zhejiang University School of Medicine, Hangzhou, China

**Keywords:** Biomarkers, Epidemiology, Frailty, Immune function

## Abstract

**Background:**

The neutrophil-to-lymphocyte ratio (NLR), platelet-to-lymphocyte ratio (PLR) and systemic immune-inflammation index (SII) are readily available circulatory immunity markers that are associated with components of frailty. However, few studies have investigated the relationship between these immunity markers and frailty, and it remains unknown whether they are predictive of incident frailty in older adults in general. Hence, we aimed to examine the association of these immunity markers with the risk of incident frailty.

**Results:**

Overall, 1822 older adults (mean age was 78.03 ± 4.46 years) were included in the Rugao Longitudinal Aging Study. NLR, PLR and SII were calculated from blood cell counts. The frailty definition was based on the Fried phenotype. At baseline, 200 (10.98%) individuals were defined as frailty, and no significant associations of NLR, PLR and SII with frailty were found. During the 2-year follow-up, 180 (15.67%) individuals were new-onset frailty. After adjustment, an increased logNLR (odds ratio [OR] 2.92, 95% confidence interval [CI] 1.20–7.18), logPLR (OR 2.54, 95% CI: 1.01–6.53) and logSII (OR 2.34, 95% CI: 1.16–4.78) were significantly associated with a higher risk of incident frailty in all individuals. Additionally, the associations of logNLR (OR 4.21, 95% CI 1.54–11.62 logPLR (OR 3.38, 95% CI: 1.17–9.91) and logSII (OR 2.56, 95% CI: 1.15–5.72) with incident frailty were remained after excluding individuals with comorbidities. In further analyzed, individuals with higher levels of NLR and SII had higher risk of incident frailty when we stratified individuals by quartiles of these immunity markers.

**Conclusion:**

NLR and SII are easily obtained immunity markers that could be used to predict incident frailty in clinical practice.

**Supplementary Information:**

The online version contains supplementary material available at 10.1186/s12979-021-00257-6.

## Background

Frailty, a prominent phenotype of accelerated aging, is characterized by a loss of physiologic reserve and resistance to stressors due to cumulative declines in many physiological systems throughout the life course [1]. The prevalence of frailty is approximately 12% among individuals aged 50+ years [2]. Additionally, frailty is a significant risk factor for adverse outcomes, such as disability [3], multi-morbidity [4] and mortality [5]. It has become an emerging global health burden with major implications for clinical practice and public health [6]. While individuals with frailty are able to dynamically transition between states, it is important to identify the high-risk population with frailty and then prevent them from developing adverse outcomes.

Age-associated changes in the immune system (characterized as a decline in immune function and an increase in low-grade, chronic systemic inflammation) have been suggested to be associated with frailty [7–9]. Neutrophils are important biomarkers of innate immunity, and platelets may contribute to immune function, whereas lymphocytes potentially reveal massive information about adaptive immunity [10]. A combination of these immune markers, including the neutrophil-to-lymphocyte ratio (NLR), platelet-to-lymphocyte ratio (PLR) and systemic immune-inflammation index (SII), is thought to better reflect alterations in the immune system [11, 12]. Evidence from observational studies has demonstrated a significant association of these circulatory markers of immunity with increased risks of dementia [13], cardiovascular disease [14] and mortality [15, 16] in older adults. Meanwhile, several studies have indicated a significant association of immune markers with frailty in specific populations, such as patients with coronary heart disease [17]. Additionally, associations of immune markers with precursor syndrome and/or components of frailty (including a slow gait speed and sarcopenia) have been reported [18–20]. However, in the general elderly population, few studies have investigated the association of these circulatory immunity markers with frailty, and it remains unknown whether they are predictive of incident frailty.

Therefore, to understand the association of these immunity markers (NLR, PLR and SII) with the risk of developing frailty, we first hypothesized that NLR, PLR and SII would be associated with frailty and then determined the longitudinal association of these immunity markers with the risk of incident frailty in older adults in general.

## Methods

### Study population

**Fig. 1:**
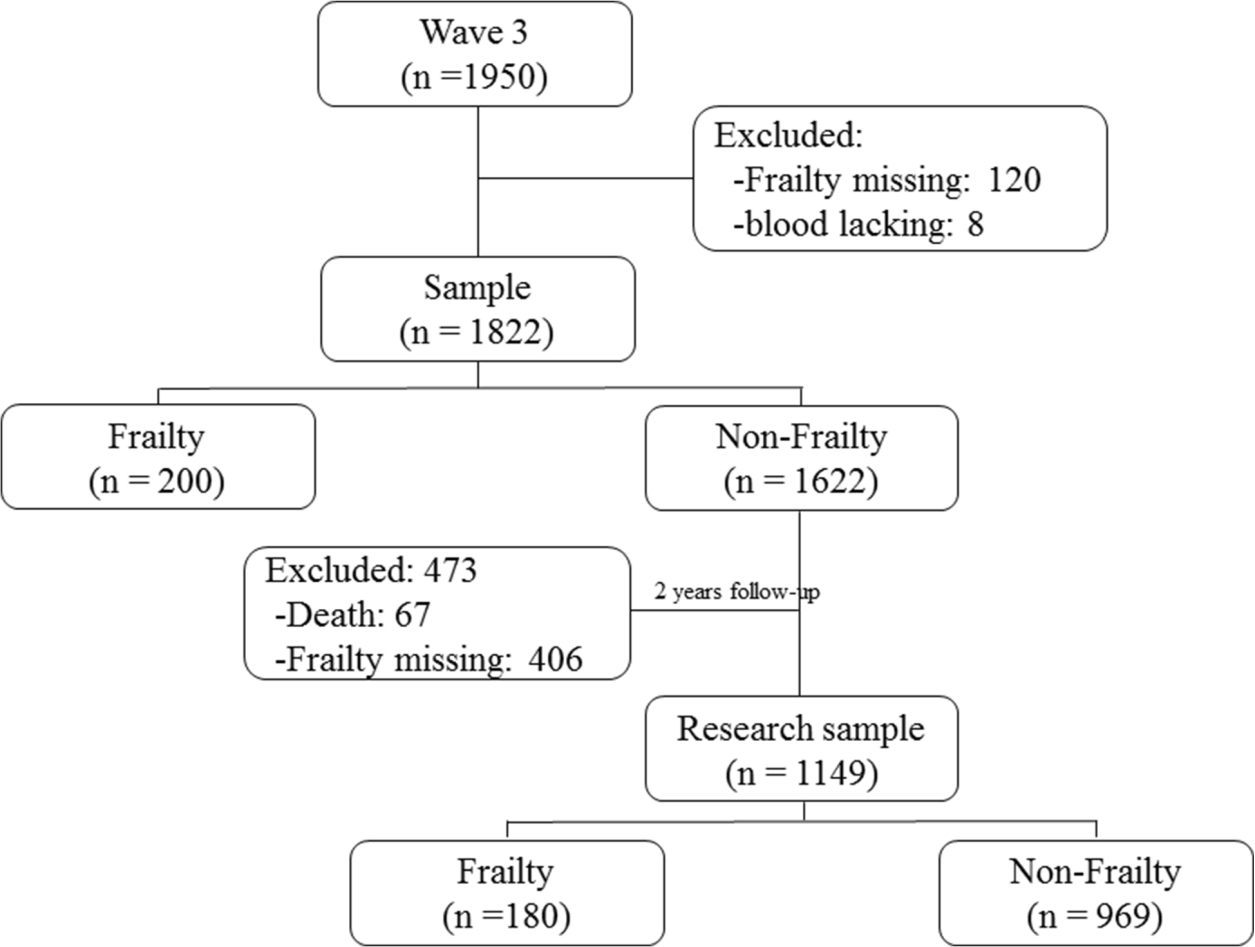
Flowchart of the study population

The Rugao Longitudinal Aging Study (RLAS) was an observational, prospective and community-based cohort study [21]. The first survey was conducted from November to December 2014 (wave 1). Then, the second survey was conducted in April 2016 (wave 2). The third and fourth surveys were conducted in November 2017 (wave 3) and December 2019 (wave 4), respectively. In the current study, the third wave was recognized as the baseline, and 1950 older adults were recruited. Among these participants, 120 individuals lacked frailty data, and 8 individuals lacked blood samples. In addition, 200 individuals were diagnosed as frailty. Hence, 1622 individuals were followed up during the subsequent 2 years. During the follow-up period, 67 participants died, and 406 participants lacked complete frailty information and/or withdrew from the study. Finally, 1149 participants were included and analyzed in this longitudinal setting (Fig. 1). The Human Ethics Committee of the School of Life Sciences of Fudan University, Shanghai, China, approved this study (No: BE1815). Written consent was obtained from all participants prior to the study.

### Outcomes

#### Fried’s frailty phenotype

The assessment of frailty status was conducted at baseline and at the 2-year follow-up. According to the frailty phenotype by Fried and colleagues, five criteria (including weight loss, weakness, exhaustion, slowness and low activity) were used to define frailty [22]. As previously reported [23], weight loss, exhaustion and low activity were measured by self-reported items. In detail, weight loss was defined if the participant responded “yes” to the question “Have you lost more than 4.5 kg or 5% of your body weight in the past 12 months?” Exhaustion was defined if the participant responded “yes” to the question “Have you felt tired at least 3 or 4 days per week?” Low activity was considered if the participant responded “yes” to the question: “Do you need help to walk?” Slowness was defined as being below the 20th percentile in the timed ‘up and go’ test (TUG). In the TUG, participants are asked to stand up from an armchair, walk 3 m, return, and sit down again. The timing of the test begins when the participant’s back is removed from the back of the chair and it stops when their buttocks touch the seat of the chair at the end of the test. Weakness was defined as being below the 20th specific percentile in maximum handgrip strength using a dynamometer (grip force, Shanghai Wanqing Electronics Co., Ltd. Shanghai, China) for three trials of each hand. The maximum value of the two hands was used in this study. Participants with any three or more indicators were defined as frail, one or two as ‘prefrail’ and none as‘robust’ [22].

#### Measurements of immunity markers

Blood samples were collected, and full blood count measurements were performed immediately after the blood draw. These measurements (including absolute counts of neutrophils, platelets and lymphocytes) were performed using an Olympus AU5811 clinical chemistry analyzer (Tokyo, Japan) with standard laboratory techniques. The neutrophil-to-lymphocyte ratio (NLR) was calculated based on the absolute neutrophil count (N; × 10^9^/L) and lymphocyte (L; × 10^9^/L) blood counts (NLR = N/L). The platelet-to-lymphocyte ratio (PLR) was calculated for the absolute peripheral platelet (P; × 10^9^/L) and lymphocyte blood counts (PLR = P/L). The systemic immune-inflammation index (SII) was calculated for the platelets and the NLR (SII = P × NLR) [11]. The NLR, PLR and SII were nonnormally distributed and were therefore log-transformed prior to performing the analyses.

#### Covariates

Demographic and clinical characteristics, laboratory data and lifestyles were collected. The demographic data included age, gender, marital status and educational status. Specifically, participants who married and lived together were categorized into the married group, while those who never married or were divorced, separated or widowed were assigned to the other group. Education status included illiteracy (never attended any school) and nonilliteracy. The clinical characteristics included self-reported cardiovascular disease (CVD), cancer and self-reported hypertension. Self-reported CVD included cerebral infarction, stroke, cerebral hemorrhage, coronary heart disease, myocardial infarction and heart failure. Comorbidities included self-reported CVD and cancer. Laboratory data included high-density lipoprotein (HDL), low-density lipoprotein (LDL), fasting blood glucose (FBG) and triglyceride (TG) levels. Lifestyles included smoking, alcohol consumption, regular exercise and body mass index (BMI). In brief, current and former smokers were assigned to the smoking group, while never smokers were categorized into no smoking groups; similarly, participants who self-reported current and former alcohol drinking were assigned to the drinking group, while those with never drinking were categorized into the no drinking group. Regular exercise was assessed by asking participants if their leisure time physical activity was more frequent than three times per week. The participants who responded “Yes” were recognized as engaging in regular exercise.

### Statistical analysis

First, the characteristics of the study population were described at baseline. We also divided the NLR, PLR and SII into four groups according to the quartiles (Q). For NLR, Q1: ≤ 1.26; Q2: > 1.26, ≤1.71; Q3: > 1.71, ≤2.35; Q4: > 2.35 (Supplemental Table S1). For PLR, Q1: ≤ 71.07; Q2: > 71.07, ≤93.79; Q3: > 93.79, ≤126.81; Q4: > 126.81 (Supplemental Table S2). For SII: Q1: ≤ 213.61; Q2: > 213.61, ≤309.45; Q3: > 309.45, ≤445.76; Q4: > 445.76 (Supplemental Table S3). Then, we described the different characteristics of the study population between the quartiles of NLR, PLR and SII. Continuous and categorical variables are presented as the mean with standard deviation or frequency (%), respectively. Group differences were analyzed by chi-square or ANOVA. Second, logistic regression models were conducted to evaluate the association of these inflammatory markers (NLR, PLR and SII) with frailty in all individuals. Third, to exclude the influences of comorbidities, these logistic regression models were reconstructed after excluding individuals with comorbidities. All analyses were conducted in two models. Model 1: unadjusted, Model 2: adjusted for age, gender, BMI, smoking, alcohol consumption, education, marital status, regular exercise, self-reported hypertension, triglyceride, HDL, LDL, FBG, frailty status at baseline (of longitudinal analyses) and comorbidity (of all individuals). A *p* value (two-tailed) less than 0.05 was considered statistically significant. All analyses were conducted with SPSS 22.0 and R (version 3.6.1: www.r-project.org/).

## Results

### Characteristics of the study population

In our study, a total of 1822 (504 women) individuals were included and analyzed in cross-sectional setting, and their detailed information is shown in Table 1. Their mean age was 78.03 ± 4.46 years. Their mean NLR, PLR and SII were 1.97 ± 1.12, 104.26 ± 50.56 and 378.13 ± 295.56, respectively. During the 2-year follow-up period, 180 (15.67%) individuals were recognized as new-onset frailty.

**Table 1:** **Characteristics of the study population stratified by gender at baseline**

Characteristics		Total (*N* = 1822)	Males (*n* = 848)	Females (*n* = 974)	*P*-value
Age	M ± SD, years	78.03 ± 4.46	77.92 ± 4.44	78.13 ± 4.47	0.300
Body mass index	M ± SD, kg/m^2^	24.14 ± 6.04	23.86 ± 3.31	24.39 ± 7.65	0.062
Smoking ^ǂ^	Yes, n (%)	365 (20.03%)	327 (38.56%)	38 (3.90%)	< 0.001
	No, n (%)	1440 (79.03%)	510 (63.80%)	930 (95.48%)	
Alcohol assumption ^ǂ^	Yes, n (%)	553 (29.25%)	381 (44.93%)	172 (17.66%)	< 0.001
	No, n (%)	1246 (68.39%)	453 (53.42%)	793 (81.41%)	
Educational status ^ǂ^	Illiteracy	879 (48.24%)	148 (17.45%)	731 (75.05%)	< 0.001
	Non-illiteracy	942 (51.70%)	699 (82.43%)	243 (24.95%)	
Marital status^&, ǂ^	Married, n (%)	1171 (64.27%)	627 (73.94%)	544 (55.85%)	< 0.001
	Others ^&^, n (%)	607 (33.32%)	201 (23.70%)	406 (41.68%)	
Regular exercise ^ǂ^	Yes, n (%)	412 (22.61%)	238 (28.07%)	174 (17.86%)	< 0.001
	No, n (%)	1237 (67.56%)	531 (62.62%)	706 (72.48%)	
Triglyceride	M ± SD, mmol/L	1.42 ± 1.01	1.22 ± 0.88	1.59 ± 1.09	< 0.001
High-density lipoprotein	M ± SD, mmol/L	1.85 ± 0.45	1.84 ± 0.45	1.86 ± 0.46	0.371
Low-density lipoprotein	M ± SD, mmol/L	2.80 ± 0.63	2.70 ± 0.61	2.88 ± 0.64	< 0.001
Fasting blood glucose	M ± SD, mmol/L	5.83 ± 1.50	5.73 ± 1.36	5.92 ± 1.60	0.006
Neutrophil count	M ± SD, ×10^9^/L	3.53 ± 1.36	3.65 ± 1.42	3.43 ± 1.29	0.001
Lymphocytes count	M ± SD, × 10^9^/L	2.05 ± 0.94	2.00 ± 1.11	2.09 ± 0.77	0.036
Platelet count	M ± SD, × 10^9^/L	190.57 ± 67.78	182.11 ± 61.25	197.97 ± 72.22	< 0.001
NLR	M ± SD	1.97 ± 1.12	2.12 ± 1.27	1.84 ± 0.96	< 0.001
PLR	M ± SD	104.26 ± 50.56	103.01 ± 50.05	105.37 ± 51.01	0.319
SII	M ± SD	378.13 ± 295.56	389.32 ± 306.74	368.56 ± 285.39	0.135
Self-report hypertension	Yes, n (%)	848 (46.54%)	371 (43.75%)	477 (48.97%)	0.015
	No, n (%)	974 (53.46%)	477 (56.25%)	497 (51.03%)	0.265
Comorbidity ^Ŧ^	Yes, n (%)	341 (18.72%)	153 (18.04%)	188 (19.30%)	0.269
	No, n (%)	1481 (81.28%)	695 (81.96%)	786 (80.70%)	
Frailty status at baseline	Robust, n (%)	583 (32.00%)	333 (39.27%)	250 (25.66%)	< 0.001
	Pre-Frailty, n (%)	1039 (57.02%)	452 (53.30%)	587 (60.27%)	
	Frailty, n (%)	200 (10.98%)	63 (7.43%)	137 (14.07%)	

M: mean, SD: standard deviation. NLR: neutrophil-to-lymphocytes ratio, PLR: platelet-to-lymphocyte ratio, SII: systemic immune-inflammation index, Continuous and categorical variables were present as mean with SD and frequency (%), Group difference were analyzed by chi-square or ANOVA test. ^&^ including separated, divorced, never married or widowed. ^ǂ^ Unknown: Educational status (1, 0.05%), Marital status (44, 2.41%), Alcohol assumption (23, 1.26%), Smoking: (17, 0.93%), Regular exercise: (173, 9.50%). ^Ŧ^ Including cerebral infarction, stroke, cerebral hemorrhage, coronary heart disease, myocardial infarction, heart failure and cancer

### Cross-sectional association of the NLR, PLR and SII with frailty

Supplemental Table S4 showed the cross-sectional association of the NLR, PLR and SII with frailty. There was significant association of logNLR (odds ratio [OR] 2.94, 95% confidence interval [CI] 1.33–6.48) and logSII (OR 2.12, 95% CI 1.13–3.98) with frailty were found in all individuals after adjusted for confounders. While, after excluded individuals with comorbidities, there were no significant association between these immunity marker and frailty (for logNLR: OR 2.35, 95% CI 0.92–5.99; for logPLR: OR 1.82, 95% CI 0.69–4.88; for logSII: OR 1.79, 95% CI 0.85–3.76). Meanwhile, similarly results were found in further analyzed when we stratified individuals by quartiles of these immunity marker in with (Supplemental Table S5) and without comorbidities (Supplemental Table S 6).

### Longitudinal association between the NLR and incident frailty

The associations of the NLR with incident frailty were explored in longitudinal settings. After controlling for confounding factors, there was significant association of increased logNLR (OR 2.92, 95% CI 1.20–7.18, *P*-value = 0.019) with incident frailty (Table 2). Individuals in higher NLR group (Q4) had higher risk (OR 2.39, 95% CI 1.39–4.21, *P*-value = 0.002) of incident frailty (Table 3).

**Table 2:** Association of immunity markers with risk of incident frailty in non-frail individuals

	Model 1		Model 2	
	OR (95% CI)	*P*-value	OR (95% CI)	*P*-value
**All individuals**				
NLR	2.14 (0.98, 4.67)	0.056	2.92 (1.20, 7.18)	0.019
PLR	2.72 (1.18, 6.34)	0.019	2.54 (1.01, 6.53)	0.050
SII	2.14 (1.14, 4.03)	0.018	2.34 (1.16, 4.78)	0.019
Neutrophil count	1.58 (0.54, 4.64)	0.406	2.49 (0.72, 8.72)	0.151
Platelet count	2.39 (0.81, 7.36)	0.122	1.70 (0.51, 6.00)	0.398
Lymphocytes count	0.34 (0.12, 0.99)	0.048	0.30 (0.09, 1.00)	0.051
**Without comorbidities**				
NLR	3.13 (1.31, 7.47)	0.010	4.21 (1.54, 11.62)	0.005
PLR	3.58 (1.39, 9.30)	0.009	3.38 (1.17, 9.91)	0.025
SII	2.50 (1.23, 5.09)	0.012	2.56 (1.15, 5.72)	0.021
Neutrophil count	1.73 (0.51, 5.89)	0.376	2.33 (0.56, 9.76)	0.244
Platelet count	2.03 (0.60, 7.19)	0.261	1.32 (0.33, 5.47)	0.697
Lymphocytes count	0.18 (0.06, 0.59)	0.005	0.14 (0.04, 0.54)	0.004

NLR: neutrophil-lymphocytes ratio, PLR: platelet-to-lymphocyte ratio, SII: systemic immune-inflammation index, OR: odds ratio, CI: confidence interval, Model 1: unadjusted, Model 2: adjusted for age, gender, BMI, smoking, alcohol assumption, educational status, marital status, regular exercise, elf-reported hypertension, triglyceride, high-density lipoprotein, low-density lipoprotein, fasting blood glucose, and comorbidity (in all individuals). All immunity markers were logarithmically transformed. Analysis for each blood cell type adjusted for the baseline blood cell counts of the remaining two blood cell types

**Table 3:** Association of immunity markers with risk of incident frailty in all individuals

Immunity markers	Event/N (%)	Model 1		Model 2	
		OR (95% CI)	*P*-value	OR (95% CI)	*P*-value
NLR	180/1149 (15.67%)				
Q1 (≤ 1.26)	41/292 (14.04%)	Ref	Ref	Ref	Ref
Q2 (> 1.26, ≤1.71)	44/312 (14.10%)	1.00 (0.63, 1.59)	0.995	1.16 (0.67, 2.03)	0.590
Q3 (> 1.71, ≤2.35)	43/279 (15.41%)	1.12 (0.70, 1.78)	0.644	1.33 (0.77, 2.33)	0.309
Q4 (> 2.35)	52/266 (19.55%)	1.49 (0.95, 2.34)	0.082	2.39 (1.39, 4.21)	0.002
PLR	180/1149 (15.67%)				
Q1 (≤ 71.07)	39/287 (13.59%)	Ref	Ref	Ref	Ref
Q2 (> 71.07, ≤93.79)	46/317 (14.51%)	1.08 (0.68, 1.72)	0.745	0.98 (0.57, 1.71)	0.954
Q3 (> 93.79, ≤126.81)	45/285 (15.79%)	1.19 (0.75, 1.89)	0.468	1.08 (0.63, 1.84)	0.787
Q4 (> 126.81)	50/260 (19.23%)	1.51 (0.96, 2.40)	0.075	1.35 (0.79, 2.32)	0.273
SII	180/1149 (15.67%)				
Q1 (≤ 213.61)	38/295 (12.88%)	Ref	Ref	Ref	Ref
Q2 (> 213.61, ≤309.45)	45/307 (14.66%)	1.16 (0.73, 1.85)	0.538	1.37 (0.78, 2.44)	0.276
Q3 (> 309.45, ≤445.76)	46/293 (15.70%)	1.23 (0.77, 1.97)	0.380	1.55 (0.89, 2.73)	0.126
Q4 (> 445.76)	51/254 (20.08%)	1.70 (1.08, 2.70)	0.024	2.30 (1.32, 4.08)	0.004

NLR: neutrophil-lymphocytes ratio, PLR: platelet-to-lymphocyte ratio, SII: systemic immune-inflammation index, OR: odds ratio, CI: confidence interval, Model 1: unadjusted, Model 2: adjusted for age, gender, BMI, smoking, alcohol assumption, educational status, marital status, regular exercise, self-report hypertension, triglyceride, high-density lipoprotein, low-density lipoprotein, fasting blood glucose and comorbidity

In addition, we further reanalyzed these associations between the NLR level and incident frailty among individuals without comorbidities (Table 4). With a 1-unit increase in the logNLR, the OR for incident frailty was 4.21 (95% CI 1.54–11.62, *P*-value = 0.005) after controlling for confounding factors (Table 2). Meanwhile, Individuals in higher NLR group (Q4) had higher risk (OR 2.88, 95% CI 1.54–5.55, *P*-value = 0.001) of incident frailty (Table 4).

**Table 4:** Association of immunity markers with risk of incident frailty in individuals without comorbidities

Immunity markers	Event/N (%)	Model 1		Model 2	
		OR (95% CI)	*P*-value	OR (95% CI)	*P*-value
NLR	142/956 (14.85%)				
Q1 (≤ 1.26)	24/232 (10.34%)	Ref	Ref	Ref	Ref
Q2 (> 1.26, ≤1.71)	35/257 (13.62%)	1.36 (0.79, 2.39)	0.275	1.20 (0.63, 2.33)	0.580
Q3 (> 1.71, ≤2.35)	39/241 (16.18%)	1.67 (0.98, 2.92)	0.064	1.80 (0.95, 3.45)	0.073
Q4 (> 2.35)	44/226 (19.47%)	2.10 (1.24, 3.63)	0.007	2.88 (1.54, 5.55)	0.001
PLR	142/956 (14.85%)				
Q1 (≤ 71.07)	27/231 (11.69%)	Ref	Ref	Ref	Ref
Q2 (> 71.07, ≤93.79)	38/267 (14.23%)	1.25 (0.74, 2.14)	0.401	1.17 (0.62, 2.22)	0.637
Q3 (> 93.79, ≤126.81)	36/236 (15.25%)	1.35 (0.79, 2.33)	0.268	1.23 (0.66, 2.33)	0.514
Q4 (> 126.81)	41/222 (18.47%)	1.71 (1.02, 2.92)	0.045	1.56 (0.84, 2.95)	0.167
SII	142/956 (14.85%)				
Q1 (≤ 213.61)	26/231 (11.26%)	Ref	Ref	Ref	Ref
Q2 (> 213.61, ≤309.45)	33/258 (12.79%)	1.15 (0.67, 2.00)	0.614	1.32 (0.67, 2.65)	0.420
Q3 (> 309.45, ≤445.76)	39/247 (15.79%)	1.44 (0.85, 2.48)	0.181	1.73 (0.90, 3.40)	0.104
Q4 (> 445.76)	44/220 (20.00%)	1.97 (1.17, 3.37)	0.011	2.59 (1.39, 5.00)	0.003

NLR: neutrophil-lymphocytes ratio, PLR: platelet-to-lymphocyte ratio, SII: systemic immune-inflammation index, OR: odds ratio, CI: confidence interval, Model 1: unadjusted, Model 2: adjusted for age, gender, BMI, smoking, alcohol assumption, educational status, marital status, regular exercise, self-reported hypertension, triglyceride, high-density lipoprotein, low-density lipoprotein and fasting blood glucose

### Longitudinal association between the PLR and incident frailty

In crude models, with a 1-unit increase in the logPLR, the risk for incident frailty was increased to 2.72 (95% CI: 1.18–6.34, P-value = 0.19) (Table 2). Similarly, consistent results were observed after adjusted for confounder factors in individuals with (OR 2.54, 95% CI 1.01–6.53, *P*-value = 0.050) and without comorbidities (OR 3.38, 95% CI 1.17–9.91, *P*-value = 0.025). In addition, we stratified individuals by quartiles of NLR in further analyzed. However, no significant associations were found in individuals with (Table 3) and without comorbidities (Table 4).

### Longitudinal association between the SII and incident frailty

We first investigated the association of immune markers with frailty in all individuals. We found that the risk of incident frailty was 2.14 (95% CI: 1.14–4.03, *P*-value = 0.018) with a 1-unit logSII increase in the crude model (Table 2). Individuals in the quartile 4 group had a higher risk of incident frailty (OR = 1.70, 95% CI: 1.08–2.70, *P*-value = 0.024) than those in the lowest SII group (Table 3). After adjustments, the association of SII with the risk of incident frailty remained in the quartile 4 groups (OR = 2.30, 95% CI: 1.32–4.08, *P*-value = 0.004), and individuals with increased logSII had an increased risk of incident frailty (OR = 2.34, 95% CI: 1.16–4.78, P-value = 0.019). Additionally, we further analyzed these associations of the SII with the risk of incident frailty among individuals without comorbidities (Tables 2 and 4). Similar results were found for the SII levels and incident frailty in the crude (per unit of logSII: OR = 2.50, 95% CI: 1.23–5.09, P-value = 0.012) and adjusted models (per unit of logSII: OR = 2.56, 95% CI: 1.15–5.72, P-value = 0.021).

## Discussion

### Principal findings

In this longitudinal study, we investigated the predictive effect of circulatory markers of immunity for incident frailty in older adults. Our principal findings were that an elevated NLR and SII were robustly associated with an increased risk of incident frailty. These associations were even stronger in individuals without comorbidities. Additionally, because NLR and SII are extensively and readily obtainable in the laboratory and clinical fields, they may be proposed as predictors for the incidence of frailty.

### Compared with other studies

To date, several studies have examined the association of immune markers with frailty by studying the NLR, PLR and SII in older adults in general. Collerton et al. conducted a cross-sectional study in the Newcastle 85+ Study and found inverse associations of memory/naïve CD8 T and B cell ratios with the frailty index and physical frailty [24]. In addition, several studies suggested a significant association of the NLR, PLR and SII levels with precursor syndrome and/or components of frailty. Gait speed is a feasible predictor of health-risk assessment in geriatrics [25–27] and is considered a hallmark of frailty. Previous studies reported a significant association between NLR levels and slow gait speed in older adults in general (OR = 3.82, 95% CI: 1.87–7.89, *P*-value < 0.001) [18]. Additionally, similar significant associations were also found between the NLR level and a slow gait speed in patients with cancer (Spearman’s correlation coefficient [r]: - 0.48, *P* value =0.0001) [28] and coronary heart disease (r: 0.211, *P*-value =0.001) [17], respectively.

Sarcopenia is often considered a precursor syndrome or a physical component of frailty. Several studies have explored associations between these immunity markers and sarcopenia. Zhao et al. included 4224 middle-aged and older adults from the West China Health and Aging Trend (WCHAT) study and examined these associations in a cross-sectional cohort. They found that a higher NLR (OR = 1.123, 95% CI: 1.047–1.205, *P*-value < 0.01), PLR (OR = 1.004, 95% CI: 1.003–1.006, P-value < 0.001) and SII (OR = 1.001, 95% CI: 1.000–1.001, P-value < 0.001) were associated with an increased risk of sarcopenia [19]. Similarly, Öztürk et al. included 419 individuals and reported a significant association of NLR with sarcopenia in a case-control study (OR = 1.31, 95% CI: 1.06–1.62, P-value =0.013) [20]. Liaw et al. also enrolled 3671 individuals aged ≥60 years from the National Health and Nutrition Examination Survey (NHANES) III and found that elevated PLR levels were significantly associated with the risk of sarcopenia (OR = 1.001, 95% CI: 1.007–1.009, P-value < 0.001) [29]. In brief, previous studies showed that these immunity markers are associated with precursor syndromes and/or components of frailty, which is consistent with our findings that these immunity markers are associated with the risk of incident frailty in older adults in general.

### The possible mechanisms

The mechanisms of the association of NLR and SII with incident frailty are not entirely known. Several pathways are involved in the association of the altered immune system with frailty. Neutrophils are important biomarkers of innate immunity, and platelets may contribute to immune function, whereas lymphocytes potentially reveal massive information on adaptive immunity [10]. NLR, PLR and SII are thought to reflect the balance between the innate and adaptive immune systems [11, 12]. While, age-associated changes in the immune system could also lead to an increase in low-grade, chronic systemic inflammation [30]. NLR, PLR and SII are proposed as inflammatory biomarkers. Inflammation is widely recognized to be associated with frailty, such as for other inflammatory markers like C-reactive protein, interleukin-6 and tumor necrosis factor α [31–33]. The potential mechanism may be that inflammation is associated with reduced synthesis and activity of insulin-like growth factor I (IGF-I), which is essential for muscle regeneration and the maintenance of muscle integrity [34]. A loss of skeletal muscle strength and mass is a precursor syndrome and/or component of frailty.

### Strengths and limitations

Several strengths exist in this study. First, we examined and validated the association of the NLR and SII levels with incident frailty in prospective, longitudinal and community-based cohorts. The generalization and reliability of our findings could be improved. Second, to validate our hypothesis, we conducted our study after adjusting for potential confounding factors that could affect these circulatory markers of immunity (such as BMI, physical activity and smoking) [35–37] and analyzed the association of these immunity markers with incident frailty in individuals with and without comorbidities. These sensitivity analyses attempted to rule out the potential effects of confounding factors. Third, these immunity markers (NLR and SII) obtained from complete blood counts are inexpensive, easy to measure and are reproducible in the laboratory. More importantly, due to the government of China providing free routine medical examinations for elderly individuals every year at community health service stations [38], these immunity markers could be developed annually by utilizing pre-existing annual routine medical examination data, not placing any extra burdens on the local government, community doctors or older adults.

The limitations were also presented in our study. Because of NLR, PLR and SII were measured at one point; subsequent temporal changes in these immunity markers were not captured. Therefore, longitudinal studies with repeated measurements during the follow-up may mitigate these limitations, improve the reliability, and illustrate the natural history of exposure-induced outcomes. In addition, we had not conducted any measurements on immune cell function or assessed systemic inflammation with markers such as CRP, interleukins 6 and interleukins 1, and then much of this really does not relate to their biomarkers. Further studies would focus on exploring possible relationship between immune function and frailty in the future.

## Conclusion

In conclusion, in this community-based study, we found that higher NLR and SII were associated with an increased risk of incident frailty in older adults in general. The clinical implications are that these immunity markers convey massive information on the future incidence of frailty and further reveal the importance of conducting interventions to improve the immune system to limit the incidence of frailty in older adults. Additionally, since NLR and SII levels are extensively used in the clinical field and are readily available assessments in the laboratory, they may be proposed as cost-effective predictors for the future incidence of frailty. More importantly, benefitting from free routine medical examinations in China, these immunity markers could be inspected annually without any additional medical burdens and should be used to conveniently predict the risk of frailty.

## Supplementary Information

**Additional file 1: Supplemental Table S1.** Characteristics of the study population stratified by quartiles of NLR levels at baseline. **Supplemental Table S2.** Characteristics of the study population stratified by quartiles of PLR levels at baseline. **Supplemental Table S3.** Characteristics of the study population stratified by quartiles of SII levels at baseline. **Supplemental Table S4.** Association of immunity markers with risk of frailty at baseline. **Supplemental Table S5.** Association of immunity markers with risk of frailty among all individuals at baseline. **Supplemental Table S6**. Association of immunity markers with risk of frailty among individuals without comorbidity at baseline

## Data Availability

The datasets generated and/or analyzed during the current study are available from the corresponding author on reasonable request.

## Abbreviations

NLR: neutrophil-to-lymphocyte ratio
PLR: platelet-to-lymphocyte ratio
SII: systemic immune-inflammation index
OR: odds ratio
CI: confidence interval
RLAS: The Rugao Longitudinal Aging Study
TUG: ‘up and go’ test
CVD: cardiovascular disease
HDL: high-density lipoprotein
LDL: low-density lipoprotei
FBG: fasting blood glucose
TG: triglyceride
BMI: body mass index
Q: quartiles
