# Supplementary material for: Association of immunity markers with the risk of incident frailty: the Rugao longitudinal aging study
Source: Immun Ageing. 2022 Jan 3;19:1. doi: 10.1186/s12979-021-00257-6 (PMC8722120; doi:10.1186/s12979-021-00257-6)
Supplement: Supplementary file 1 — Additional file 1: Supplemental Table S1. Characteristics of the study population stratified by quartiles of NLR levels at baseline. Supplemental Table S2. Characteristics of the study population stratified by quartiles of PLR levels at baseline. Supplemental Table S3. Characteristics of the study population stratified by quartiles of SII levels at baseline. Supplemental Table S4. Association of immunity markers with risk of frailty at baseline. Supplemental Table S5. Association of immunity markers with risk of frailty among all individuals at baseline. Supplemental Table S6. Association of immunity markers with risk of frailty among individuals without comorbidity at baseline [file 12979_2021_257_MOESM1_ESM.docx]

**Supplemental Table S1. Characteristics of the study population stratified by quartiles of NLR levels at baseline.**

| **Characteristics** | **Subgroups** | **Q1**  **(≤ 1.26)** | **Q2**  **(>1.26, ≤1.71)** | **Q3**  **(>1.71, ≤2.35)** | **Q4**  **(>2.35)** | **P-value** |
| --- | --- | --- | --- | --- | --- | --- |
| No. | 1822 | 450 | 462 | 450 | 460 |  |
| Age | M ± SD, years | 77.99 ± 4.46 | 77.81 ± 4.56 | 78.30 ± 4.50 | 78.03 ± 4.46 | 0.434 |
| Gender ^ǂ^ | Males, n (%) | 177 (39.33%) | 203 (43.94%) | 208 (46.22%) | 260 (56.52%) | < 0.001 |
|  | Females, n (%) | 273 (60.67%) | 259 (56.06%) | 242 (53.78%) | 200 (43.48%) |  |
| Body mass index | M ± SD, kg/m^2^ | 24.55 ± 10.39 | 23.74 ± 3.39 | 24.42 ± 3.71 | 23.83 ± 3.70 | 0.109 |
| Smoking ^ǂ^ | Yes, n (%) | 75 (16.67%) | 94 (20.35%) | 94 (20.89%) | 102 (22.17%) | 0.209 |
|  | No, n (%) | 369 (82.00%) | 365 (79.00%) | 351 (78.00%) | 355 (77.17%) |  |
| Alcohol assumption ^ǂ^ | Yes, n (%) | 143 (31.78%) | 151 (32.68%) | 123 (27.33%) | 136 (29.57%) | 0.314 |
|  | No, n (%) | 301 (66.89%) | 307 (66.45%) | 321 (71.33%) | 317 (68.91%) |  |
| Educational status ^ǂ^ | Illiteracy | 238 (52.89%) | 217 (46.97%) | 207 (46.00%) | 217 (47.17%) | 0.154 |
|  | Non-illiteracy | 212 (47.11%) | 244 (52.81%) | 243 (54.00%) | 243 (52.83%) |  |
| Marital status^&, ǂ^ | Married, n (%) | 281 (62.44%) | 291 (62.99%) | 297 (66.00%) | 302 (65.65%) | 0.772 |
|  | Others ^&^, n (%) | 156 (34.67%) | 156 (33.77%) | 145 (32.22%) | 150 (32.61%) |  |
| Regular exercise ^ǂ^ | Yes, n (%) | 95 (21.11%) | 107 (23.16%) | 104 (23.11%) | 106 (23.04%) | 0.865 |
|  | No, n (%) | 308 (67.78%) | 310 (67.10%) | 297 (66.00%) | 322 (70.00%) |  |
| Triglyceride | M ± SD, mmol/L | 1.43 ± 0.98 | 1.45 ± 1.01 | 1.37 ± 0.88 | 1.42 ± 1.16 | 0.688 |
| HDL | M ± SD, mmol/L | 1.90 ± 0.48 | 1.84 ± 0.44 | 1.82 ± 0.44 | 1.84 ± 0.45 | 0.054 |
| LDL | M ± SD, mmol/L | 2.86 ± 0.63 | 2.83 ± 0.63 | 2.76 ± 0.64 | 2.74 ± 0.62 | 0.016 |
| FBG, | M ± SD, mmol/L | 5.80 ± 1.46 | 5.72 ± 1.28 | 5.93 ± 1.66 | 5.87 ± 1.55 | 0.168 |
| Neutrophil count | M ± SD, ×10^9^/L | 2.60 ± 0.70 | 3.13 ± 0.82 | 3.69 ± 1.03 | 4.70 ± 1.64 | < 0.001 |
| Lymphocytes count | M ± SD, ×10^9^/L | 2.75 ± 1.38 | 2.12 ± 0.56 | 1.86 ± 0.53 | 1.47 ± 0.47 | < 0.001 |
| Platelet count | M ± SD, ×10^9^/L | 189.02 ± 56.60 | 190.69 ± 63.39 | 189.83 ± 71.50 | 192.69 ± 77.77 | 0.864 |
| NLR | M ± SD | 0.99 ± 0.20 | 1.48 ± 0.13 | 2.00 ± 0.18 | 3.38 ± 1.32 | < 0.001 |
| PLR | M ± SD | 74.08 ± 26.21 | 94.46 ± 36.57 | 106.98 ± 40.26 | 140.97 ± 64.75 | < 0.001 |
| SII | M ± SD | 187.16 ± 69.82 | 282.17 ± 96.60 | 379.53 ± 149.17 | 659.93 ± 431.33 | < 0.001 |
| Hypertension | Yes, n (%) | 201 (44.67%) | 205 (44.37%) | 217 (48.22%) | 225 (48.91%) | 0.382 |
|  | No, n (%) | 249 (55.33%) | 257 (55.63%) | 233 (51.78%) | 235 (51.09%) |  |
| Comorbidity ^Ŧ^ | Yes, n (%) | 95 (21.11%) | 88 (19.05%) | 74 (16.44%) | 84 (18.26%) | 0.345 |
|  | No, n (%) | 355 (78.89%) | 374 (80.95%) | 376 (83.56%) | 376 (81.74%) |  |
| Frailty status at baseline | Robust, n (%) | 158 (35.11%) | 152 (32.90%) | 148 (32.89%) | 125 (27.17%) | 0.111 |
|  | Pre-Frailty, n (%) | 249 (55.33%) | 265 (57.36%) | 244 (54.22%) | 281 (61.09%) |  |
|  | Frailty, n (%) | 43 (9.56%) | 45 (9.74%) | 58 (12.89%) | 54 (11.74%) |  |

NLR: neutrophil-lymphocytes ratio, HDL: High-density lipoprotein, Low-density lipoprotein, FBG: Fasting blood glucose, M: mean, SD: standard deviation. NLR: Neutrophil to lymphocytes ratio, Continuous and categorical variables were present as mean with SD and frequency (%), Group difference were analyzed by chi-square or ANOVA test. ^&^ including separated, divorced, never married or widowed. ^ǂ^ Unknown: Educational status (1, 0.05%), Marital status (44, 2.41%), Alcohol assumption (23, 1.26%), Smoking: (17, 0.93%), Regular exercise: (173, 9.50%). ^Ŧ^ Including cerebral infarction, stroke, cerebral hemorrhage, coronary heart disease, myocardial infarction, heart failure and cancer.

**Supplemental Table S2. Characteristics of the study population stratified by quartiles of PLR levels at baseline.**

| **Characteristics** | **Subgroups** | **Q1**  **(≤ 71.07)** | **Q2**  **(>71.07, ≤93.79)** | **Q3**  **(>93.79, ≤126.81)** | **Q4**  **(>126.81)** | **P-value** |
| --- | --- | --- | --- | --- | --- | --- |
| No. | 1822 | 456 | 455 | 455 | 456 |  |
| Age | M ± SD, years | 78.05 ± 4.47 | 77.73 ± 4.41 | 77.87 ± 4.46 | 78.46 ± 4.48 | 0.072 |
| Gender ^ǂ^ | Males, n (%) | 228 (50.00%) | 213 (46.81%) | 208 (45.71%) | 199 (43.64%) | 0.275 |
|  | Females, n (%) | 228 (50.00%) | 242 (53.19%) | 247 (54.29%) | 257 (56.36%) |  |
| Body mass index | M ± SD, kg/m^2^ | 24.63 ± 10.24 | 24.41 ± 3.76 | 24.16 ± 3.72 | 23.35 ± 3.50 | 0.008 |
| Smoking ^ǂ^ | Yes, n (%) | 103 (22.59%) | 83 (18.24%) | 81 (17.80%) | 98 (21.49%) | 0.206 |
|  | No, n (%) | 349 (76.54%) | 368 (80.88%) | 367 (80.66%) | 356 (78.07%) |  |
| Alcohol assumption ^ǂ^ | Yes, n (%) | 147 (32.23%) | 131 (28.79%) | 141 (30.99%) | 134 (29.39%) | 0.621 |
|  | No, n (%) | 302 (66.23%) | 319 (70.11%) | 307 (67.47%) | 318 (69.74%) |  |
| Educational status ^ǂ^ | Illiteracy | 207 (45.39%) | 212 (46.59%) | 222 (48.79%) | 238 (52.19%) | 0.185 |
|  | Non-illiteracy | 249 (54.61%) | 242 (53.19%) | 233 (51.21%) | 218 (47.81%) |  |
| Marital status^&, ǂ^ | Married, n (%) | 295 (64.69%) | 301 (66.15%) | 294 (64.62%) | 281 (61.62%) | 0.402 |
|  | Others ^&^, n (%) | 149 (32.68%) | 142 (31.21%) | 149 (32.75%) | 167 (36.62%) |  |
| Regular exercise ^ǂ^ | Yes, n (%) | 114 (25.00%) | 106 (23.30%) | 101 (22.20%) | 91 (19.96%) | 0.264 |
|  | No, n (%) | 301 (66.01%) | 297 (65.27%) | 314 (69.01%) | 325 (71.27%) |  |
| Triglyceride | M ± SD, mmol/L | 1.57 ± 1.17 | 1.44 ± 0.96 | 1.32 ± 1.00 | 1.34 ± 0.88 | 0.001 |
| HDL | M ± SD, mmol/L | 1.80 ± 0.45 | 1.88 ± 0.48 | 1.87 ± 0.44 | 1.87 ± 0.45 | 0.023 |
| LDL | M ± SD, mmol/L | 2.76 ± 0.63 | 2.83 ± 0.65 | 2.80 ± 0.61 | 2.79 ± 0.63 | 0.425 |
| FBG, | M ± SD, mmol/L | 1.52 ± 0.07 | 1.63 ± 0.08 | 1.42 ± 0.06 | 1.40 ± 0.06 | 0.261 |
| Neutrophil count | M ± SD, ×10^9^/L | 3.43 ± 1.16 | 3.46 ± 1.31 | 3.49 ± 1.35 | 3.75 ± 1.56 | 0.001 |
| Lymphocytes count | M ± SD, ×10^9^/L | 2.68 ± 1.40 | 2.19 ± 0.59 | 1.86 ± 0.47 | 1.44 ± 0.44 | < 0.001 |
| Platelet count | M ± SD, ×10^9^/L | 141.79 ± 45.77 | 178.88 ± 47.67 | 202.76 ± 49.19 | 238.85 ± 81.26 | < 0.001 |
| NLR | M ± SD | 1.41 ± 0.59 | 1.66 ± 0.71 | 1.97 ± 0.93 | 2.81 ± 1.49 | < 0.001 |
| PLR | M ± SD | 54.87 ± 12.09 | 81.90 ± 6.39 | 109.50 ± 9.75 | 170.73 ± 51.47 | < 0.001 |
| SII | M ± SD | 188.77 ± 78.47 | 282.85 ± 107.15 | 382.80 ± 157.14 | 657.89 ± 428.81 | < 0.001 |
| Hypertension | Yes, n (%) | 214 (46.93%) | 205 (45.05%) | 209 (45.93%) | 220 (48.25%) | 0.794 |
|  | No, n (%) | 242 (53.07%) | 250 (54.95%) | 246 (54.07%) | 236 (51.75%) |  |
| Comorbidity ^Ŧ^ | Yes, n (%) | 97 (21.27%) | 81 (17.80%) | 90 (19.78%) | 73 (16.01%) | 0.192 |
|  | No, n (%) | 359 (78.73%) | 374 (82.20%) | 365 (80.22%) | 383 (83.99%) |  |
| Frailty status at baseline | Robust, n (%) | 139 (30.48%) | 165 (36.26%) | 151 (33.19%) | 128 (28.07%) | 0.074 |
|  | Pre-Frailty, n (%) | 266 (58.33%) | 253 (55.60%) | 252 (77.36%) | 268 (58.77%) |  |
|  | Frailty, n (%) | 51 (11.18%) | 37 (8.13%) | 52 (11.43%) | 60 (13.16%) |  |

NLR: neutrophil-lymphocytes ratio, HDL: High-density lipoprotein, Low-density lipoprotein, FBG: Fasting blood glucose, M: mean, SD: standard deviation. NLR: Neutrophil to lymphocytes ratio, Continuous and categorical variables were present as mean with SD and frequency (%), Group difference were analyzed by chi-square or ANOVA test. ^&^ including separated, divorced, never married or widowed. ^ǂ^ Unknown: Educational status (1, 0.05%), Marital status (44, 2.41%), Alcohol assumption (23, 1.26%), Smoking: (17, 0.93%), Regular exercise: (173, 9.50%). ^Ŧ^ Including cerebral infarction, stroke, cerebral hemorrhage, coronary heart disease, myocardial infarction, heart failure and cancer.

**Supplemental Table S3. Characteristics of the study population stratified by quartiles of SII levels at baseline.**

| **Characteristics** | **Subgroups** | **Q1**  **(≤ 213.61)** | **Q2**  **(>213.61, ≤309.45)** | **Q3**  **(>309.45, ≤445.76)** | **Q4**  **(>445.76)** | **P-value** |
| --- | --- | --- | --- | --- | --- | --- |
| No. | 1822 | 456 | 455 | 455 | 456 |  |
| Age | M ± SD, years | 77.86 ± 4.45 | 77.91 ± 4.44 | 78.06 ± 4.62 | 78.29 ± 4.46 | 0.457 |
| Gender ^ǂ^ | Males, n (%) | 207 (45.39%) | 208 (45.71%) | 215 (47.25%) | 218 (47.81%) | 0.861 |
|  | Females, n (%) | 249 (54.61%) | 247 (54.29%) | 240 (52.75%) | 238 (52.19%) |  |
| Body mass index | M ± SD, kg/m^2^ | 24.25 ± 10.28 | 24.22 ± 3.51 | 24.37 ± 3.83 | 23.71 ± 3.63 | 0.369 |
| Smoking ^ǂ^ | Yes, n (%) | 89 (19.52%) | 90 (19.78%) | 95 (20.88%) | 91 (19.96%) | 0.944 |
|  | No, n (%) | 363 (79.61%) | 362 (79.56%) | 353 (77.58%) | 362 (79.39%) |  |
| Alcohol assumption ^ǂ^ | Yes, n (%) | 143 (31.36%) | 143 (31.43%) | 151 (33.19%) | 116 (25.44%) | 0.067 |
|  | No, n (%) | 305 (66.89%) | 308 (67.69%) | 300 (65.93%) | 333 (73.03%) |  |
| Educational status ^ǂ^ | Illiteracy | 219 (48.03%) | 220 (48.35%) | 210 (46.15%) | 230 (50.44%) | 0.639 |
|  | Non-illiteracy | 237 (51.97%) | 234 (51.43%) | 245 (53.85%) | 226 (49.56%) |  |
| Marital status^&, ǂ^ | Married, n (%) | 294 (64.47%) | 301 (66.15%) | 275 (60.44%) | 301 (66.01%) | 0.219 |
|  | Others ^&^, n (%) | 151 (33.11%) | 142 (31.21%) | 169 (37.14%) | 145 (31.80%) |  |
| Regular exercise ^ǂ^ | Yes, n (%) | 105 (23.03%) | 98 (21.54%) | 109 (23.96%) | 100 (21.93%) | 0.890 |
|  | No, n (%) | 303 (66.45%) | 305 (67.03%) | 312 (68.57%) | 317 (69.52%) |  |
| Triglyceride | M ± SD, mmol/L | 1.40 ± 1.01 | 1.40 ± 0.95 | 1.45 ± 1.17 | 1.42 ± 0.92 | 0.835 |
| HDL | M ± SD, mmol/L | 1.88 ± 0.49 | 1.87 ± 0.46 | 1.83 ± 0.44 | 1.83 ± 0.44 | 0.198 |
| LDL | M ± SD, mmol/L | 2.75 ± 0.64 | 2.81 ± 0.63 | 2.77 ± 0.60 | 2.86 ± 0.65 | 0.054 |
| FBG, | M ± SD, mmol/L | 5.70 ± 1.28 | 5.85 ± 1.48 | 5.91 ± 1.70 | 5.86 ± 1.48 | 0.173 |
| Neutrophil count | M ± SD, ×10^9^/L | 2.59 ± 0.75 | 3.19 ± 0.85 | 3.69 ± 1.00 | 4.66 ± 1.66 | < 0.001 |
| Lymphocytes count | M ± SD, ×10^9^/L | 2.44 ± 1.43 | 2.14 ± 0.68 | 1.96 ± 0.60 | 1.64 ± 0.60 | < 0.001 |
| Platelet count | M ± SD, ×10^9^/L | 142.86 ± 44.63 | 178.03 ± 48.97 | 200.54 ± 48.42 | 240.84 ± 81.18 | < 0.001 |
| NLR | M ± SD | 1.17 ± 0.42 | 1.59 ± 0.52 | 1.97 ± 0.59 | 3.13 ± 1.46 | < 0.001 |
| PLR | M ± SD | 63.50 ± 21.06 | 87.61 ± 25.19 | 107.57 ± 29.91 | 158.34 ± 58.10 | < 0.001 |
| SII | M ± SD | 155.17 ± 41.51 | 260.66 ± 27.92 | 371.79 ± 39.98 | 724.61 ± 401.92 | < 0.001 |
| Hypertension | Yes, n (%) | 189 (41.45%) | 196 (43.08%) | 242 (53.19%) | 221 (48.46%) | 0.001 |
|  | No, n (%) | 267 (36.62%) | 259 (56.92%) | 213 (46.81%) | 235 (51.54%) |  |
| Comorbidity ^Ŧ^ | Yes, n (%) | 101 (22.15%) | 89 (19.56%) | 78 (17.14%) | 73 (16.01%) | 0.083 |
|  | No, n (%) | 355 (77.85%) | 366 (80.44%) | 377 (82.86%) | 383 (83.99%) |  |
| Frailty status at baseline | Robust, n (%) | 153 (33.55%) | 165 (36.26%) | 144 (31.65%) | 121 (26.54%) | 0.006 |
|  | Pre-Frailty, n (%) | 252 (55.26%) | 247 (54.29%) | 272 (59.78%) | 268 (58.77%) |  |
|  | Frailty, n (%) | 51 (11.18%) | 43 (9.45%) | 39 (8.57%) | 67 (14.69%) |  |

NLR: neutrophil-lymphocytes ratio, HDL: High-density lipoprotein, Low-density lipoprotein, FBG: Fasting blood glucose, M: mean, SD: standard deviation. NLR: Neutrophil to lymphocytes ratio, Continuous and categorical variables were present as mean with SD and frequency (%), Group difference were analyzed by chi-square or ANOVA test. ^&^ including separated, divorced, never married or widowed. ^ǂ^ Unknown: Educational status (1, 0.05%), Marital status (44, 2.41%), Alcohol assumption (23, 1.26%), Smoking: (17, 0.93%), Regular exercise: (173, 9.50%). ^Ŧ^ Including cerebral infarction, stroke, cerebral hemorrhage, coronary heart disease, myocardial infarction, heart failure and cancer.

**Supplemental Table S4 Association of immunity markers with risk of frailty at baseline**

|  | Model 1 |  | Model 2 |  |
| --- | --- | --- | --- | --- |
|  | OR (95% CI) | P-value | OR (95% CI) | P-value |
| **All individuals** | |  |  |  |
| NLR | 1.87 (0.93, 3.72) | 0.077 | 2.94 (1.33, 6.48) | 0.008 |
| PLR | 2.05 (0.98, 4.35) | 0.059 | 2.27 (0.99, 5.31) | 0.056 |
| SII | 1.74 (1.00, 3.04) | 0.050 | 2.12 (1.13, 3.98) | 0.020 |
| Neutrophil count | 1.49 (0.56, 3.96) | 0.422 | 2.47 (0.81, 7.51) | 0.111 |
| Platelet count | 1.71 (0.64, 4.71) | 0.294 | 1.28 (0.44, 3.90) | 0.655 |
| Lymphocytes count | 0.44 (0.17, 1.13) | 0.087 | 0.29 (0.10, 0.85) | 0.024 |
| **Without comorbidities** | |  |  |  |
| NLR | 1.44 (0.64, 3.21) | 0.375 | 2.35 (0.92, 5.99) | 0.729 |
| PLR | 1.63 (0.69, 3.88) | 0.267 | 1.82 (0.69, 4.88) | 0.229 |
| SII | 1.52 (0.79, 2.89) | 0.205 | 1.79 (0.85, 3.76) | 0.126 |
| Neutrophil count | 1.39 (0.44, 4.34) | 0.576 | 2.12 (0.56, 8.01) | 0.266 |
| Platelet count | 1.72 (0.54, 5.65) | 0.362 | 1.15 (0.32, 4.22) | 0.831 |
| Lymphocytes count | 0.68 (0.23, 2.02) | 0.490 | 0.39 (0.11, 1.37) | 0.141 |

NLR: neutrophil-lymphocytes ratio, PLR: platelet-to-lymphocyte ratio, SII: systemic immune-inflammation index, OR: odds ratio, CI: confidence interval, Model 1: unadjusted, Model 2: adjusted for age, gender, BMI, smoking, alcohol assumption, educational status, marital status, regular exercise, elf-reported hypertension, triglyceride, high-density lipoprotein, low-density lipoprotein, fasting blood glucose and comorbidities (in all individuals). All immunity markers were logarithmically transformed. Analysis for each blood cell type adjusted for the baseline blood cell counts of the remaining two blood cell types.

**Supplemental Table S5 Association of immunity markers with risk of frailty among all individuals at baseline**

| Immunity markers | Event/N (%) | Model 1 |  | Model 2 |  |
| --- | --- | --- | --- | --- | --- |
|  |  | OR (95% CI) | P-value | OR (95% CI) | P-value |
| NLR | 200/1822 (10.98%) |  |  |  |  |
| Q1 (≤1.26) | 43/450 (9.56%) | Ref | Ref | Ref | Ref |
| Q2 (>1.26, ≤1.71) | 45/462 (9.74%) | 1.02 (0.66, 1.59) | 0.925 | 0.93 (0.55, 1.58) | 0.790 |
| Q3 (>1.71, ≤2.35) | 58/450 (12.89%) | 1.40 (0.92, 2.14) | 0.114 | 1.55 (0.96, 2.53) | 0.072 |
| Q4 (>2.35) | 54/460 (11.74%) | 1.26 (0.83, 1.93) | 0.287 | 1.42 (0.88, 2.31) | 0.152 |
| PLR | 200/1822 (10.98%) |  |  |  |  |
| Q1 (≤ 71.07) | 51/456 (11.18%) | Ref | Ref | Ref | Ref |
| Q2 (>71.07, ≤93.79) | 37/455 (8.13%) | 0.70 (0.45, 1.09) | 0.120 | 0.58 (0.34, 0.99) | 0.043 |
| Q3 (>93.79, ≤126.81) | 52/455 (11.43%) | 1.02 (0.68, 1.55) | 0.907 | 1.00 (0.62, 1.59) | 0.991 |
| Q4 (>126.81) | 60/456 (13.16%) | 1.20 (0.81, 1.80) | 0.362 | 1.19 (0.76, 1.88) | 0.448 |
| SII | 200/1822 (10.98%) |  |  |  |  |
| Q1 (≤ 213.61) | 51/456 (11.18%) | Ref | Ref | Ref | Ref |
| Q2 (>213.61, ≤309.45) | 43/455 (9.45%) | 0.83 (0.54, 1.27) | 0.390 | 0.74 (0.45, 1.22) | 0.241 |
| Q3 (>309.45, ≤445.76) | 39/455 (8.57%) | 0.75 (0.48, 1.15) | 0.191 | 0.65 (0.39, 1.08) | 0.096 |
| Q4 (>445.76) | 67/456 (14.69%) | 1.37 (0.93, 2.03) | 0.115 | 1.37 (0.89, 2.13) | 0.158 |

NLR: neutrophil-lymphocytes ratio, PLR: platelet-to-lymphocyte ratio, SII: systemic immune-inflammation index, OR: odds ratio, CI: confidence interval, Model 1: unadjusted, Model 2: adjusted for age, gender, BMI, smoking, alcohol assumption, educational status, marital status, regular exercise, self-reported hypertension, triglyceride, high-density lipoprotein, low-density lipoprotein, fasting blood glucose and comorbidity.

**Supplemental Table S6 Association of immunity markers with risk of frailty among individuals without comorbidity at baseline**

| Immunity markers | Event/N (%) | Model 1 |  | Model 2 |  |
| --- | --- | --- | --- | --- | --- |
|  |  | OR (95% CI) | P-value | OR (95% CI) | P-value |
| NLR | 148/1481 (9.99%) |  |  |  |  |
| Q1 (≤ 1.26) | 37/355 (10.42%) | Ref | Ref | Ref | Ref |
| Q2 (>1.26, ≤1.71) | 30/374 (8.02%) | 0.75 (0.45, 1.24) | 0.263 | 0.54 (0.28, 1.00) | 0.050 |
| Q3 (>1.71, ≤2.35) | 42/376 (11.17%) | 1.08 (0.68, 1.73) | 0.745 | 1.12 (0.65, 1.93) | 0.691 |
| Q4 (>2.35) | 39/376 (10.37%) | 0.99 (0.62, 1.60) | 0.982 | 1.07 (0.63, 1.85) | 0.795 |
| PLR |  |  |  |  |  |
| Q1 (≤ 71.07) | 37/359 (10.31%) | Ref | Ref | Ref | Ref |
| Q2 (>71.07, ≤93.79) | 28/374 (7.49%) | 0.70 (0.42, 1.17) | 0.181 | 0.60 (0.32, 1.09) | 0.098 |
| Q3 (>93.79, ≤126.81) | 36/365 (9.86%) | 0.95 (0.59, 1.55) | 0.843 | 0.98 (0.56, 1.73) | 0.946 |
| Q4 (>126.81) | 47/383 (12.27%) | 1.22 (0.77, 1.93) | 0.399 | 1.29 (0.77, 2.21) | 0.339 |
| SII |  |  |  |  |  |
| Q1 (≤ 213.61) | 39/355 (10.99%) | Ref | Ref | Ref | Ref |
| Q2 (>213.61, ≤309.45) | 28/366 (7.65%) | 0.67 (0.40, 1.11) | 0.125 | 0.60 (0.33, 1.09) | 0.096 |
| Q3 (>309.45, ≤445.76) | 30/377 (7.96%) | 0.70 (0.42, 1.16) | 0.166 | 0.67 (0.37, 1.19) | 0.173 |
| Q4 (>445.76) | 51/383 (13.32%) | 1.24 (0.80, 1.95) | 0.334 | 1.25 (0.76, 2.08) | 0.387 |

NLR: neutrophil-lymphocytes ratio, PLR: platelet-to-lymphocyte ratio, SII: systemic immune-inflammation index, OR: odds ratio, CI: confidence interval, Model 1: unadjusted, Model 2: adjusted for age, gender, BMI, smoking, alcohol assumption, educational status, marital status, regular exercise, self-reported hypertension, triglyceride, high-density lipoprotein, low-density lipoprotein, and fasting blood glucose.
